# Super-swelling behavior of stacked lipid bilayer systems

**DOI:** 10.1140/epje/s10189-023-00322-6

**Published:** 2023-08-03

**Authors:** Jacob Rueben, Dylan Steer, Cecília Leal

**Affiliations:** Department of Materials Science and Engineering, University of Illinois Urbana Champaign, 1304 W. Green St. MC 246, Urbana, IL 61801 USA

## Abstract

**Abstract:**

Bilayer systems comprising lipid mixtures are the most well-studied model of biological membranes. While the plasma membrane of the cell is a single bilayer, many intra- and extra-cellular biomembranes comprise stacks of bilayers. Most bilayer stacks in nature are periodic, maintaining a precise water layer separation between bilayers. That equilibrium water separation is governed by multiple inter-bilayer forces and is highly responsive. Biomembranes re-configure inter-bilayer spacing in response to temperature, composition, or mass transport cues. In synthetic bilayer systems for applications in cosmetics or topical treatments, control of the hydration level is a critical design handle. Herein we investigate a binary lipid system that leverages key inter-bilayer forces leading to unprecedented levels of aqueous swelling while maintaining a coherent multilamellar form. We found that combining cationic lipids with bicontinuous cubic phase-forming lipids (lipids with positive Gaussian modulus), results in the stabilization of multilamellar phases against repulsive steric forces that typically lead to bilayer delamination at high degrees of swelling. Using ultra-small-angle X-ray scattering alongside confocal laser scanning microscopy, we characterized various super-swelled states of 1,2-dioleoyl-3-trimethylammonium-propane (DOTAP) and glycerol monooleate (GMO) lipids, as well as other analogous systems, at varied concentration and molar ratios. Through these experiments we established swelling profiles of various binary lipid systems that were near-linear with decreasing lipid volume fraction, showing maximum swelling with periodicity well above 200 nanometers.

**Graphical abstract:**

Confocal fluorescence micrograph of super-swelled multilamellar structures in 90GMOD sample at 25 mM concentration. Inset plot shows intensity profile of orange line, with pink triangles indicating maxima.
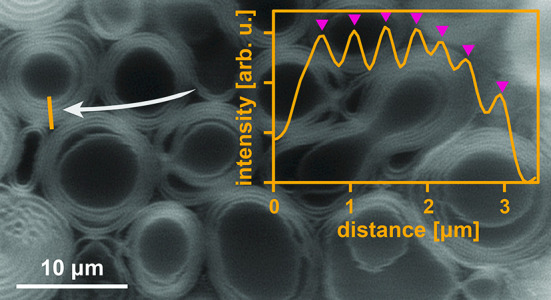

## Introduction

Lamellar liquid crystals are an extremely common subsection of semi-ordered matter often found in nature. The packing of lipids into single bilayers is a fundamental and thoroughly studied self-assembled form. Interestingly, many biomembranes in animal and plant tissue assemble into periodic stacks of bilayers. Examples of these constructs include multilamellar bodies (MLBs) in the alveolar hypophase and thylakoid membranes found inside chloroplasts and cyanobacteria. [1–3]

Multilamellar assemblies are characterized by a smectic array of lipid bilayers separated by a water layer of uniform thickness. As a response to external factors, biomembranes will adjust the number of bilayers in the stack, such as in thylakoids with fluctuating light and temperature.[4, 5] Water layer thickness can also be dynamically changed, an example being lung MLBs dehydrating as lipid composition changes due to lung injury.[6–9] The ability to mimic the adaptive hydration behavior of stacked biomembranes in synthetic multilamellar systems could bring forth a myriad of applications in pharmaceutics and cosmetics. [10–13]. For example, it has been shown that topical treatments benefit from lamellar formulations over traditional micellar/emulsive (oil-in-water) formulations due to lower toxicity and higher encapsulation power of hydrophilic and hydrophobic actives. [14] Cosmetic elegance, defined by a topical product’s ease and enjoyment of use, can also be optimized through the use of lamellar phases to alter the formulation’s stability, viscosity, and elasticity. [15] Therefore, it is important to elucidate the driving forces that control hydration of multilayers of complex lipid mixtures to unlock new design principles for lipid-based materials.

A multilamellar system can be characterized by three properties: the bilayer thickness $\delta _m$, the water layer thickness $d_w$ separating bilayers in the stack, and the lamellar repeat distance d defined as the sum of $\delta _m$ and $d_w$. [2] While lamellar lipid phases are quite common, and can persist to high repeat distances in oil-rich environments,[16–18] *d* rarely increases past a few tens of nanometers for most lipid systems in aqueous environments. [19] The $d_w$ limit is set by the equilibrium distance between bilayers in a stack emerging from a balance of inter-bilayer attractive van der Waals interactions, repulsive steric interactions (thermal undulations), and electrical double layer forces for charged lipids, allowing a narrow window of stability with respect to lamellar repeat distance. [19–22] The van der Waals force $F_{vdw}$ between flat bilayers per membrane area (*A*) is defined as:

1 $$\begin{aligned} \frac{F_{vdw}}{A}=-\frac{H}{6\pi D^3} \end{aligned}$$

Where H is the Hamaker constant, and $D=d_w$. [22] The electric double layer repulsion force per unit area is defined as:

2 $$\begin{aligned} \frac{F_{edl}}{A}=\frac{Z}{2 \pi \lambda _D^2} \exp {\left( -\frac{D}{\lambda _D}\right) } \end{aligned}$$

Where Z is a constant proportional to the charge density of the membranes and $\lambda _D$ is the Debye length.

Steiner *et al.* postulated that one can define a lipid membrane persistence length $\xi$ that evaluates the maximum lamellar repeat distance at which bilayers in a multilamellar phase remain correlated. They defined $\xi$ as:

3 $$\begin{aligned} \xi = a \exp {\left( \frac{4\pi \kappa }{3k_B T}\right) } \end{aligned}$$

Where *a* is the lipid molecule size, and $\kappa$ is the membrane bending modulus. [19] The proportional relationship between membrane bending rigidity and persistence length is supported by the inverse relationship between membrane bending modulus and undulative force per unit area of membranes at separation *D*, as:

4 $$\begin{aligned} \frac{F_{und}}{A} = \frac{3 \pi ^2 (k_B T)^2}{64 \kappa D^3} \end{aligned}$$

[23] Put simply, the conventional theory of lamellar phase stability is governed by multiple intermolecular forces, an important one being undulative repulsive forces, which become significant in low rigidity membranes. In other words, the floppier the membrane is, the easier it is to thermally activate bilayer undulations, and the lower its persistence length—and maximum lamellar repeat distance—will become. The van der Waals force brings membranes together, and lipid hydrophobic chains are preferably sequestered in the bilayer core with minimal exposure to water. In some instances, for fully saturated lipids, the enthalpic gain of close-packing alkyl chains dominates and a high-order all-trans membrane (often referred to as a gel phase) is stabilized. However, unsaturated chains loosely packed in a bilayer lead to dominant repulsive undulations resulting in membrane delamination, loss of water periodicity, and ultimately phase separation in an emulsive state. If the lipids are charged, this effect is intensified by electric double layer repulsive forces [20].

While Equation 3 references membrane bending modulus as a singular value, there are actually two separate elastic moduli used to accurately define the energy required to deform a lipid membrane. The total curvature-elastic energy of a membrane can be expressed as:

5 $$\begin{aligned} \frac{E}{A} = \frac{\kappa }{2} (J-J_0)^2 + \bar{\kappa } K \end{aligned}$$

Where the total or extrinsic curvature $J$ is a sum of the membrane’s local principal curvatures, $J = C_{1}+C_{2}$, the Gaussian curvature is $K=C_{1}C_{2}$, $J_{0}$ is the spontaneous membrane curvature, $\kappa$ is the (normal) bending modulus, and $\bar{\kappa }$ is the Gaussian curvature modulus. The Gaussian curvature modulus describes the membrane’s desire to form saddle-splay geometries with $C_1$ and $C_2$ of opposite signs. Certain lipids such as glycerol monooleate have molecular properties that favor bilayers curved into periodic minimal surfaces at $K<0$ and $J_0=0$, forming bicontinuous cubic phases [24, 25] and necessitating that $\bar{\kappa } > 0$. These phases can take up a lot of water especially if charged lipids or lipids covalently linked to polyethylene glycol are included. [26, 27] In rigid systems, Gaussian curvature considerations are less significant, as the total magnitudes of curvature are decreased. Previous studies have documented systems in this regime, with rigid lipid membranes established by high membrane charge density. [28] In this case, membranes space out ideally due to charge repulsion, until at a critical dilution factor the membranes condense into a coexisting disordered phase. The authors hypothesize that this is due in part to the negative Gaussian modulus of the charged lipids used favoring partial melting of the lamellar phase into said disordered phase.

In this paper we evaluate strategies to overcome previous limitations in lamellar swelling [19, 23, 29–38] by incorporating into the membrane lipids with positive Gaussian moduli that tend to form bicontinuous phases when pure and equilibrated in water. We hypothesized that the resultant membranes could tolerate larger amounts of deformation, being able to accommodate more water without losing stability and delaminating. We found that a binary lipid system, 90GMOD, made up of 90 mole% GMO and 10 mole% cationic DOTAP (1,2-dioleoyl-3-trimethylammonium-propane) stabilizes a multilamellar construct with record high repeat distances. We show that the effect is rather general, yielding analogous results for systems of different lipids following the same bicontinuous cubic/charged lipid formula. With this research we hope to verify previous studies of lamellar repeat distance limitations while surpassing them by creating a binary lipid framework exhibiting high degrees of aqueous swelling relevant for applications in cosmetic and pharmaceutical emulsive topical formulations.

## Methods

Glycerol monooleate and sodium chloride were purchased from Sigma-Aldrich (MO, USA). DOTAP, 18:1 EthylPC, and DOPC were purchased from Avanti Polar Lipids (AL, USA). The purchased reagents were used without any further purification. Any lipids delivered in powder form were dissolved in fresh chloroform solvent (Macron Fine Chemicals, PA, USA). Samples were prepared in quartz glass mark tubes, which were purchased from Hilgenberg GmbH (HE, DE).

### Bulk lipid sample preparation

Lipids solubilized in chloroform were mixed and vortexed to form stock solutions of each desired composition. Stock solutions were pipetted into quartz capillaries, which were left to dry at ambient temperature in a fume hood for two days. The samples were then transferred into a vacuum desiccator for at least 24 hours. Once all solvent was removed, 10 $\upmu$L of millipore water or sodium chloride solution was added to each sample, and each sample was centrifuged for one minute (Thermo Fisher Sorvall ST16R) at 3,000 rpm (1,693 rcf) to ensure all water reaches the dry lipid cake. Samples were then flame-sealed and epoxy-sealed to prevent water loss, and incubated at 45 $^{\circ }$ C for three days to encourage hydration. Samples were then left at ambient temperature for at least 24 hours before X-ray analysis.

### Ultra-small-angle X-ray scattering

Hydrated lipid samples were analyzed at beamline 12-ID-B of Argonne National Laboratory’s Advanced Photon Source. Data were collected with the beamline’s Pilatus 300K 20 Hz hybrid pixel detector (Dectris, Switzerland). The beamline used in this experiment has a photon energy of 14 keV and a beam 300 $\upmu$m wide and 20 $\upmu$m high. Some USAXS experiments were also conducted at a locally built X-ray source at the University of Illinois at Urbana-Champaign’s Materials Research Laboratory (MRL). The MRL USAXS utilizes a Xenocs GeniX3D Cu K$\alpha$ X-ray source with an energy of 8 keV, and collects data on a Pilatus 300k 20 Hz hybrid pixel detector (Dectris AG, Switzerland). 2D Data were azimuthally integrated either using Argonne’s locally authored MATLAB software, or FIT2D software from ESRF. The integrated data were then fitted in MATLAB using its included peak fitting toolbox. Gaussian fits were used, and linear backgrounds were determined on a per-scan basis.

### Confocal laser scanning microscopy

Samples for CLSM were prepared as described above, then removed from capillaries and deposited onto glass slides for imaging. We used an LSM 800 confocal microscope (Carl Zeiss Microimaging GmbH, Germany) for imaging the samples. Final images were edited and line profiles were extracted using ImageJ (National Institutes of Health).

## Results

**Fig. 1:**
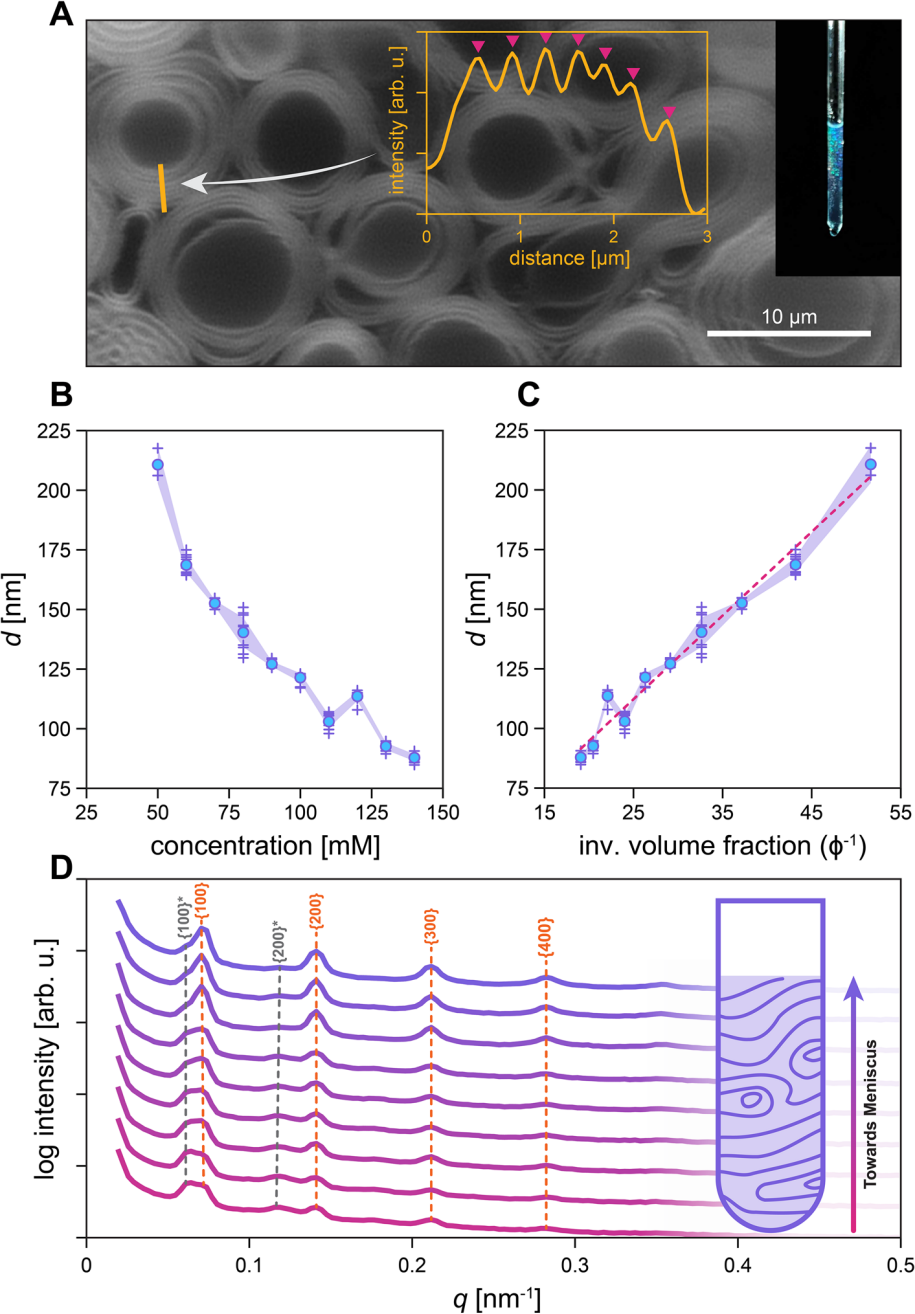
**A** Confocal fluorescence micrograph of super-swelled multilamellar structures in 90GMOD sample at 25 mM concentration. Inset plot shows intensity profile of orange line, with pink triangles indicating maxima. Overlaid image on right shows visible light diffraction from swelled structures. **B** Calculated lamellar repeat distances of 90GMOD samples, plotted against concentration. “+” symbols indicate individual data points from each vertical scan, and blue dots are averages of said scans. 95% confidence interval is shown in light purple. **C** A replotting of the data in (B), but with the lamellar repeat distances plotted against the inverse of a calculated volume fraction. A linear trend line is imposed onto the plotted data to show possible linear dependence. **D** An example dataset showing integrated USAXS data from a vertical scan of a 90GMOD sample at 130 mM concentration. Each scan was taken 4.5 mm apart, moving up the capillary towards the sample meniscus (represented by gradient of pink to purple)

We report the finding of a super-swelled multilamellar lipid phase having unusually large periodicity and coherence. This phase is made up of varying ratios of monoglyceride glycerol monooleate (GMO) and permanently charged 1,2-dioleoyl-3-trimethylammonium-propane (DOTAP), with focus placed on the composition with 90:10 GMO:DOTAP molar ratio (90GMOD). We synthesize the superswelled system through a slow evaporation-hydration process common to lipid self-assembly protocols such that the structure is likely to reach thermodynamic equilibrium. We then measure the bulk-averaged behavior of this binary lipid system, as well as analogous and dissimilar systems, using ultra-small-angle X-ray scattering (USAXS). We also inspect this system through confocal laser scanning microscopy (CLSM), a process only possible due to the uniquely swelled nature of the multilamellar structure. Through this comparative study we draw conclusions regarding the entropic origins of this phase, while relating it to current knowledge of lipid packing and molecular properties. We address the importance of this superswelled phase in a context of defining design principles for the development of new materials for biotechnological applications.

### 90GMOD dilution behavior

Figure 1B shows calculated average lamellar repeat distances from vertical USAXS scans of 90GMOD compositions with decreasing concentration. A general trend of uninhibited lamellar swelling is seen as lipid concentration is decreased from 140 mM to 50 mM, with a maximum lamellar *d*-spacing of 206 nm reached at 50 mM concentration. This data is replotted in Fig. 1C using an estimated inverse volume fraction $\phi ^{-1}$ to normalize the data accounting lipid volume. In these data a linear trend is seen between inverse volume fraction and lamellar repeat distance, evidencing a swelling profile seemingly independent of lamellar undulation magnitudes. This contradicts previous studies of purely charged, rigid lamellar systems showing a nonlinear tapering off of swelling at high $\phi ^{-1}$, purportedly due to a phase melting into a condensed disordered phase. [19]

Figure 1D shows azimuthally integrated raw data from a vertical scan of 130 mM 90GMOD, as referenced in Fig  1B-C. These data show an increasingly bimodal phase distribution with increasing distance from the sample meniscus. Farther away from the meniscus, a larger prevalence of a more swelled lamellar phase is seen, indicated by an asterisk in Fig. 1C. The vertical dependence of this more swelled phase is likely linked to evaporation at the air-water interface, hence the area near the meniscus preferring a less water-swelled phase. Both phases show clear multilamellar forms characterized by four indexed peaks of the major phase corresponding to the plane families {100}, {200}, {300}, and {400}. The less prevalent, more swelled phase shows lamellar nature as well, but only exhibiting peaks corresponding to the {100} and {200} plane families.[39]

**Fig. 2:**
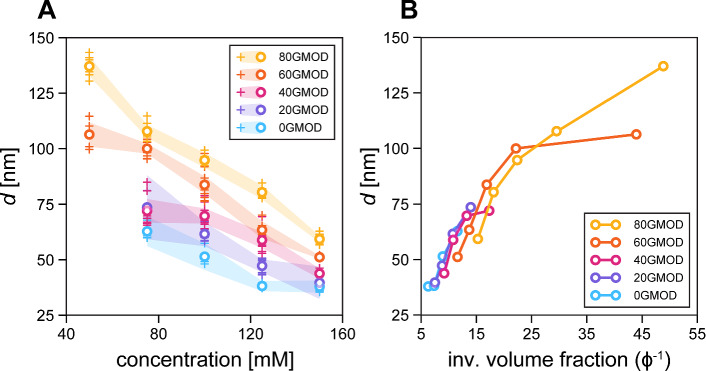
Dilution behavior of xGMOD system. **A** Concentration-dependent swelling of lipid systems with 0 mol%, 20 mol%, 40 mol%, 60 mol%, and 80 mol% GMO, the remainder being DOTAP (0GMOD, 20GMOD, 40GMOD, 60GMOD, and 80GMOD, respectively). “+” symbols indicate d-spacing calculated from single USAXS scan in vertical scan set. Open circles indicates average of said dataset. Translucent polygons indicate 95% confidence interval. **B** Averages from dataset shown in **A**, plotted against calculated inverse volume fraction

While swelling was undetectable in 90GMOD at concentrations lower than 50 mM via our USAXS setup, lamellar phases were seen at lower concentrations in CLSM imaging. Figure 1A shows a CLSM image of 25mM 90GMOD with clearly visible multilamellar vesicular structures, indicating continued order and swelling at concentrations unmeasurable using the USAXS experimental setup. A line profile was measured (orange), which shows a lamellar repeat distance of 340 nm. The inset shows a photograph of the sample quartz capillary tube showing bright scattered blue color consistent with a periodicity on the order of the wavelength of visible light.

**Fig. 3:**
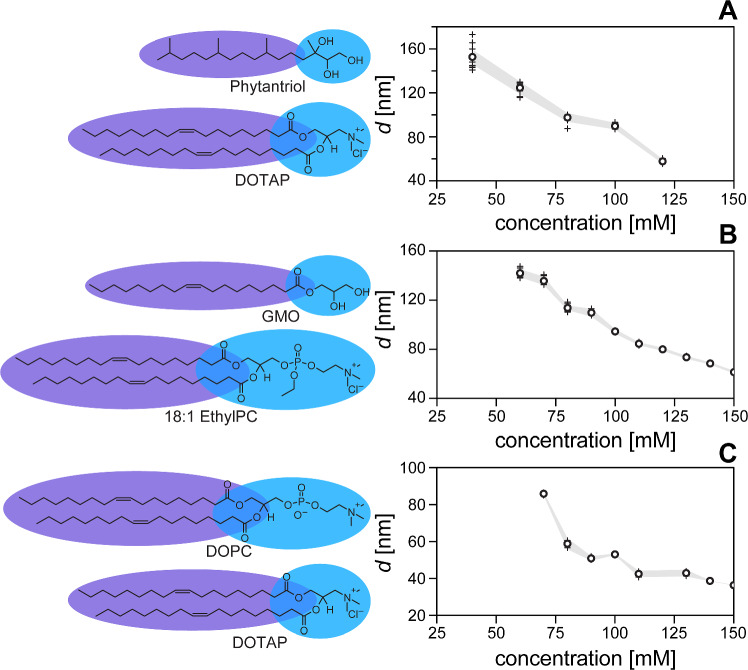
Lamellar repeat distance analysis of comparative and contrasting binary lipid systems. **A** Depictions of phytantriol (cubic lipid) and DOTAP (charged lipid), as well as lamellar repeat distance of the mixture plotted against total lipid concentration. **B** Depictions of GMO (cubic lipid) and 18:1 EthylPC (charged lipid), as well as lamellar repeat distance of the mixture plotted against total lipid concentration. **C** Depictions of DOPC (lamellar lipid) and DOTAP (charged lipid), as well as lamellar repeat distance of the mixture plotted against total lipid concentration. In Lewis diagrams, purple represents the hydrophobic region, and blue represents the hydrophilic region of the lipids used. In d-spacing plots, light gray polygons indicate 95% confidence intervals, “+” symbols indicate unaveraged d-spacings from vertical scans, and open circles indicate averaged vertical scan data

### xGMOD dilution behavior

In order to study the interplay between undulative and electrostatic forces, we tested lipid systems similar to 90GMOD, but with different ratios of charged lipid (DOTAP) to bicontinuous cubic phase forming lipid (GMO). Instead of 90 mol% GMO, compositions with 0 mol%, 20 mol%, 40 mol%, 60 mol%, and 80 mol% GMO (with remainder DOTAP) were formulated. These compositions are referred to as xGMOD, with the “x” indicating the molar percent of GMO present in the sample. Each xGMOD sample was prepared from 50 to 150 mM concentrations, with a step of 25 mM, and analyzed at various vertical positions in the capillary to obtain large sampling representation of the swelled lamellar phase. Figure 2A shows the results from this experiment plotted against concentration in a manner similar to Fig. 1B. It is evident that high degrees of swelling are seen in each xGMOD composition, with existent but not significant variation within each sample.

These data are replotted against calculated inverse volume fraction in Fig. 2B, showing significant overlap once lipid volume is normalized between xGMOD compositions. This indicates that for a given lipid volume fraction, increased charge density in the lipid membrane does not directly correlate with increased lamellar swelling in this xGMOD system. This is evidence that the swelling of the system is constrained, thereby kept from swelling further with increased electrostatic repulsion. It is also seen in Fig. 2 that while all five xGMOD compositions maintain a similar linear trend in swelling, only the 80GMOD composition was able to reach lamellar repeat distances higher than about 100 nm. Most notably, 0GMOD, 20GMOD, and 40GMOD all showed no lamellar phase at 50 mM concentration.

We argue that this perceived swelling cap is caused by the interplay of undulations and lipid curvature. With decreased molar ratio of GMO, a bicontinuous cubic phase forming lipid with positive Gaussian modulus, saddle-splay-like deformations of the membranes would become decreasingly favorable. An increasingly charged and rigid membrane bilayer conforms to previous theory and either delaminates or melts into a disordered phase, unable to swell any further. This observed phase behavior supports our hypothesis that the synergistic properties of bicontinuous cubic phase forming GMO and charged lipid DOTAP allow this super-swelling to occur.

### Dilution behavior of analogous lipid systems

To verify our hypothesis that the synergistic properties of a charged lipid and a positive-Gaussian-modulus lipid stabilize super-swelled coherent multilamellar phases, we tested the same molar ratios used in 90GMOD, while switching out both the positive-Gaussian-modulus lipid and the charged lipid. First, we switched out GMO for phytantriol, another lipid commonly used to form bicontinuous cubic phases, [40, 41] while keeping 10 mole% DOTAP as the charged component. Depictions of the chemical structure of both lipids are shown in Fig. 3A, as is the corresponding lamellar repeat distance plotted as a function of lipid concentration. One can see from the plot that the phytantriol composition also swells with a near-linear dependence on concentration, to upwards of 150 nm lamellar repeat distance. This shows that a lipid analogous to GMO can also show high degrees of swelling, decoupling the phase behavior from GMO specifically. The lack of published volumetric information on phytantriol (as well as other lipids in this subsection) prevented the calculation of lipid volume fractions as was done with the xGMOD system.

**Fig. 4:**
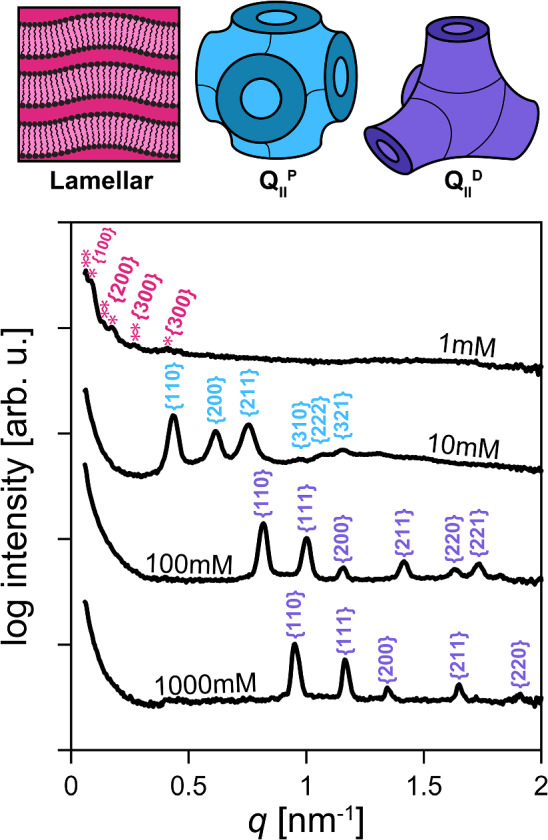
Effect of sodium chloride concentration on 90GMOD phase and lamellar repeat distance. Plot (bottom) shows example SAXS plots from 125 mM 90GMOD with increasing NaCl concentration. The peaks are indexed to plane families corresponding to the depicted liquid crystalline phases displayed (top)

For the next composition, GMO was kept as the positive-Gaussian-modulus lipid, while the charged lipid was switched out for 1,2-dioleoyl-sn-glycero-3- ethylphosphocholine (18:1 ethylPC). This is a lipid with a single positive charge, but unlike DOTAP it is an ethylated phospholipid, with similar molecular structure to that of common lamellar lipid 1,2-dioleoyl-sn-glycero-3-phosphocholine (DOPC). While DOPC is zwitterionic with no net charge, 18:1 ethylPC has a modified head group giving it a permanent positive charge. A Lewis diagram of 18:1 ethylPC is shown in Fig. 3B, as is the lamellar repeat distance plotted as a function of concentration. This composition had similar maximum observed swelling to the phytantriol-DOTAP composition, but it achieved it at a higher lipid concentration. This may have to do with different total lipid volumes in the two experiments, as each lipid has a different molecular volume in the bilayer. Regardless, 18:1 ethylPC was still able to promote high degrees of swelling, effectively demonstrating that the effect isn’t tied only to DOTAP.

The final lipid composition analyzed used DOTAP as a charged lipid, but switched out GMO for DOPC, a lipid with a negative Gaussian modulus that typically forms lamellar phases. This composition was used as a contrapositive to test whether the negative Gaussian modulus of the major lipid component was required to achieve maximum swelling. Depictions of both lipids are shown in Fig. 3C (left), and the lamellar repeat distance is plotted as a function of concentration in Fig. 3C (right). One can see from the data shown in Fig. 3C that the DOPC-DOTAP formulation shows phase instability at as high as 125 mM, where the missing data represents a lack of crystalline order detected during USAXS measurements of the capillary. At lower concentrations some order was found (shown by the scattered data points at and below 100 mM), but unlike the points shown for other compositions, these crystallites were not found consistently through the vertical scans of the respective capillaries. This further evidences the instability of the lamellar phase with the DOPC-DOTAP composition, and supports our hypothesis of the required mixture of positive-Gaussian-curvature lipid and charged lipid.

### Effects of salt on 90GMOD swelling behavior

To test the dependence of superswelled lamellar stability on charge repulsion, we conducted a salt study. We prepared 90GMOD samples of 125 mM concentration with varying amounts of sodium chloride (NaCl) in the diluent deionized water. The range from 0.01 mM to 1000 mM NaCl was tested, with steps of orders of magnitude used to probe the effects of increased charge screening, and therefore a lower Debye length, on phase behavior. Figure 4 shows the USAXS results obtained for 90GMOD equilibrated at different salt concentrations. It was found that up until 1 mM NaCl ($\lambda _D \approx$ 9.6 nm), the swelled lamellar phase was minimally affected, only decreasing from 97 to 72 nm lamellar repeat distance *d*. However, the jump from 1 to 10 mM NaCl ($\lambda _D \approx$ 3.0 nm) condensed the lamellar phase into a double diamond bicontinuous cubic phase, $Q_{II}^D$. We hypothesize that at this point, the charge repulsion caused by the DOTAP is screened enough for the undulating bilayers to interact and initiate fusion into the $Q_{II}^D$ phase. [42]. The high fusogenicity of the GMO (provided by its positive Gaussian modulus) could cause the bilayers to condense and fuse, forming the bicontinuous cubic phase. After salt concentration was increased past 100 mM ($\lambda _D \approx$ 9.6 Å), the sample changed composition once again, exhibiting a primitive bicontinuous cubic phase, $Q_{II}^P$. These results support our hypothesis that the charged lipid is required to prevent condensation of the lamellar phase, and that the charge is preventing the bicontinuous cubic phase from existing.

## Conclusions

In this study, we discovered a new design handle for creating highly swelled and coherent multilamellar lipid systems. We harness the interplay of inter-bilayer repulsive electrostatic and undulation forces with membrane curvature and elasticity. A binary system comprising cationic and neutral lipids assembled into highly flexible bilayers will swell in water to satisfy electrostatic and undulation repulsion. With regular neutral lipids like DOPC, as the water layers between bilayers swell above a certain limit, bilayers begin to delaminate and the multilamellar structure loses coherence. This swelling limit can be extended by a factor of three or more by utilizing large fractions of a neutral lipid that intrinsically tends to form bicontinuous cubic phases (having positive Gaussian modulus $\bar{\kappa }$) in pure form. Bicontinuous cubic phases comprise bilayers deformed into saddle-splays, and adding lipids with such molecular packing characteristics helps the formation of stably deformed undulatory bilayers. Combined with cationic lipids, the binary system is able to form stacks of bilayers that uptake water, maintaining periodicity without delamination. This system is most likely metastable, as evidenced by the collapse into bicontinuous cubic phases once small amounts of salt were added. Nevertheless, the bilayer stacks remain coherently hydrated at full extent over long periods of time (weeks), and the swelling behavior was replicable with multiple analogous material systems. This technique of using “non-lamellar lipids” (i.e. systems that favor nonzero Gaussian curvature) to stabilize swelling of multilamellar systems shifts the paradigm that highly rigid membranes are required to dampen repulsive protrusion forces in order to achieve stably swelled bilayer stacks.

## Data Availability

Data sets generated during the current study are available from the corresponding author on reasonable request.
